# Artificial Intelligence in Recurrent Pregnancy Loss: From Risk Prediction to ART Translation

**DOI:** 10.3390/jcm15135157

**Published:** 2026-07-02

**Authors:** Daichi Inoue

**Affiliations:** Nishitan-Art Clinic Nagoya-Ekimae, Meieki 3-28-12, Dainagoya Building 8F, Nakamura-ku, Nagoya 450-6408, Japan; my.dear.poohtan@gmail.com; Tel.: +81-52-433-8776

**Keywords:** recurrent pregnancy loss, miscarriage, artificial intelligence, machine learning, infertility, assisted reproductive technology, IVF, embryo selection, endometrial receptivity, radiomics

## Abstract

Miscarriage is a common adverse reproductive outcome, and recurrent pregnancy loss (RPL) remains a major challenge in reproductive medicine. Despite advances in genetics, immunology, endocrinology, and endometrial biology, many RPL cases remain unexplained. Conventional statistical approaches may be limited in capturing high-order nonlinear interactions among clinical, imaging, immunological, and molecular factors associated with pregnancy loss unless these interactions are explicitly modeled. Artificial intelligence (AI), including machine learning (ML) and deep learning (DL), has therefore been investigated as a potential framework for reproductive risk prediction and patient stratification. This narrative review summarizes current evidence on AI-based prediction of miscarriage and RPL, with emphasis on its possible translational relevance to infertility treatment and assisted reproductive technology (ART). Clinical data-driven models have shown potentially useful discriminatory performance, while biomarker-integrated ML approaches suggest that immune-inflammatory signatures may contribute to risk estimation. Imaging-based AI, including radiomics from multimodal ultrasound, may also support noninvasive assessment of endometrial receptivity and inform embryo transfer planning. In parallel, the broader ART literature suggests increasing interest in AI for embryo selection, embryology laboratory workflow, and ovarian stimulation prediction. However, the evidence remains limited by retrospective study designs, small datasets, inconsistent RPL definitions, inadequate external validation, and concerns regarding interpretability, fairness, and regulation. Further progress will require multimodal, explainable, and prospectively validated systems linked to clinically meaningful outcomes. AI may ultimately support more individualized reproductive care, but routine clinical implementation remains premature.

## 1. Introduction

Miscarriage is among the most common adverse outcomes in human reproduction and occurs in approximately 15–25% of clinically recognized pregnancies [1]. Recurrent pregnancy loss (RPL), depending on the definition used, affects approximately 1–2% of couples and represents a major clinical and emotional burden in reproductive medicine [1,2]. According to the updated American Society for Reproductive Medicine (ASRM) committee opinion, RPL is currently defined as two or more spontaneous pregnancy losses prior to 22 weeks of gestation, with hCG-confirmed biochemical losses counted toward the diagnosis and with confirmed molar or ectopic pregnancies excluded [3]. The updated European Society of Human Reproduction and Embryology (ESHRE) guideline adopts a comparable two-or-more-loss threshold, reflecting a broader trend toward earlier evaluation and intervention [4]. It is important to note, however, that the AI studies cited throughout this review use heterogeneous operational definitions (for example, Liu and Dong [5] used two consecutive miscarriages before 20 weeks of gestation, whereas Bruno et al. [6] stratified patients into four risk classes by number of miscarriages within an Italian RPL Unit population). These entities are not interchangeable, and apparent performance differences between studies must be interpreted with this definitional heterogeneity in mind. Table 1 summarizes the operational RPL definitions used by the major guidelines and by the principal AI studies cited in this review.

Despite substantial advances in reproductive genetics, immunology, endocrinology, and endometrial biology, the underlying cause of nearly half of RPL cases remains unknown [9]. This persistent diagnostic gap underscores the limitations of reductionist approaches and highlights the need for analytical methods capable of integrating heterogeneous clinical and biological data [9]. At the same time, infertility treatment and assisted reproductive technology (ART) increasingly depend on multidimensional decision-making involving patient characteristics, ovarian response, embryo development, endometrial status, and laboratory workflow. Within this broader context, artificial intelligence (AI) has emerged as a potentially relevant tool in reproductive medicine [9,10,11,12,13,14].

Machine learning (ML) and deep learning (DL) have already been explored across assisted reproduction, including IVF outcome prediction, embryo selection, embryology laboratory automation, and ovarian stimulation modeling [10,11,12,13,14]. Systematic reviews suggest that AI may outperform or complement embryologist-based embryo assessment, although external validation and prospective clinical implementation remain limited [12]. Recent reviews have also emphasized the importance of explainability, with a gradual shift from opaque “black-box” systems toward more interpretable “glass-box” models [13]. These developments are directly relevant to miscarriage and RPL prediction, where emotionally consequential decisions require not only predictive performance but also clinical interpretability.

In miscarriage and RPL research, AI offers an opportunity to move beyond isolated risk factors toward more integrative reproductive phenotyping. This is particularly relevant because RPL is not a single disease entity but a heterogeneous syndrome involving overlapping maternal, embryonic, immunologic, endocrine, anatomic, thrombotic, and environmental contributors [9,15]. Several representative studies have illustrated the feasibility of this approach. Bruno et al. developed an ML-based stratification model using expanded clinical data from women with RPL [6]. Liu and Dong reported prediction models incorporating inflammatory and immune biomarkers [5], and Yan et al. showed that radiomics combined with multimodal ultrasound and ML could identify endometrial features associated with unexplained RPL [7]. Additional studies in early pregnancy ultrasound and embryo assessment suggest that miscarriage prediction may also have relevance for ART-related decision-making before embryo transfer or during early gestational follow-up [16,17,18,19,20].

While a recent narrative review by Zikopoulos et al. [9] has thoughtfully summarized the current AI-in-RPL evidence base, the present review is intended to complement rather than duplicate that work. Three distinguishing features motivate this article. First, our framing is explicitly translational and oriented to the assisted reproductive technology (ART) clinical pathway: rather than treating AI in RPL as an isolated research field, we structure the discussion around its insertion points along the pathway (preconception work-up, ovarian stimulation, embryo selection, endometrial receptivity assessment, embryo transfer, early-pregnancy monitoring, and live birth), with attention to the relationships among miscarriage prediction, euploid loss, PGT-A, and male/paternal-factor variables. Second, we move beyond reported discrimination metrics (AUC, accuracy) toward an explicit appraisal of calibration, decision-curve benefit, predictive values at relevant prevalence, external validity, and patient outcomes, illustrated through worked examples from the cited studies. Third, we devote dedicated paragraphs to the molecules, genes, and immune-cell populations nominated by ML/DL studies, situating these findings within the broader single-cell architecture of the maternal-fetal interface so that mechanistic insight, rather than algorithmic novelty, is the unit of comparison across studies.

The aim of this narrative review is to summarize the current evidence on AI-based prediction of miscarriage and RPL, organized by major data source and modeling strategy, and to discuss how these approaches may contribute to infertility treatment and ART-oriented precision reproductive medicine.

## 2. Materials and Methods

This narrative review was prepared in accordance with the SANRA (Scale for the Assessment of Narrative Review Articles) framework [21]. A structured literature search of PubMed/MEDLINE was conducted through June 2026, supplemented by reference-list screening of recent systematic and narrative reviews [2,9] and by targeted searches on Google Scholar to identify peer-reviewed primary studies not retrieved by the principal database. Search terms included combinations of “recurrent pregnancy loss”, “recurrent miscarriage”, “early pregnancy loss”, “miscarriage”, “artificial intelligence”, “machine learning”, “deep learning”, “neural network”, “radiomics”, “endometrial receptivity”, “embryo selection”, “preimplantation genetic testing”, and “ovarian stimulation”. The search was restricted to English-language, peer-reviewed publications, with no lower date limit. Studies were screened by the author against pre-specified eligibility criteria: a study was included when it (i) applied ML or DL to predict miscarriage or recurrent pregnancy loss; (ii) used ML/DL to identify biomarkers, transcriptomic signatures, immune-cell populations, or molecular pathways implicated in RPL; or (iii) addressed an ART-relevant AI application with direct implications for RPL management (including embryo selection, endometrial-receptivity assessment, ovarian stimulation, or PGT-A integration). Editorials, conference abstracts without full peer-reviewed reports, and non-English publications were excluded. From each included study, data on population characteristics, RPL or miscarriage definition, sample size, modeling strategy, validation approach, discrimination, calibration where reported, and reported limitations were extracted. Given the narrative nature of this review, no formal risk-of-bias assessment was performed; instead, methodological strengths and limitations are discussed thematically throughout, with explicit reference to TRIPOD+AI and DECIDE-AI reporting standards [22,23] in Section 7.

Generative artificial intelligence (Claude 4, Anthropic, San Francisco, CA, USA; Claude Opus 4 series, accessed via https://claude.ai during 2026) was used to assist with literature search, identification and verification of candidate primary references, drafting of selected paragraphs, and copy-editing of the manuscript. All AI-generated content was critically reviewed, verified against the cited primary sources, and edited by the author, who takes full responsibility for the scientific accuracy and final wording of the manuscript. No patient-level data or original images were generated by AI tools.

## 3. Clinical Data and AI-Based Risk Stratification

Clinical data remain the most widely used input source for AI models in miscarriage and RPL research. Commonly used variables include maternal age, body mass index, number of previous pregnancy losses, endocrine profiles, autoimmune markers, thrombophilia-related factors, uterine abnormalities, and reproductive history [9]. This structured data environment is well suited to classical ML approaches and may be attractive for early clinical translation because many of these variables are already available in routine reproductive care.

The multifactorial nature of RPL has been illustrated by expanded diagnostic work-up studies. Ticconi et al. evaluated 843 women with RPL using 44 diagnostic factors grouped into seven major categories and found that 78.17% of patients had more than one abnormality [15]. The mean number of pregnancy losses increased with the number of abnormalities detected, supporting the view that RPL often reflects cumulative reproductive burden rather than a single isolated cause [15]. Such findings provide a rationale for AI-based risk modeling that can integrate multiple partially informative predictors.

Bruno et al. were among the first to apply ML to this problem in a clinically interpretable manner [6]. Using data from 734 women, they developed a support vector machine (SVM) model to stratify patients with RPL into four risk classes based on miscarriage history. Using the full set of 43 clinical variables, the model achieved a balanced accuracy of 81.86 ± 0.35%; a reduced 18-feature model performed similarly, with a balanced accuracy of 81.71 ± 0.37% [6]. By contrast, a model restricted to ESHRE-recommended features showed substantially lower performance, with a balanced accuracy of 58.52 ± 0.58% [6]. These findings suggest that broader phenotyping may be more informative than a minimal work-up alone and that AI may help translate expanded diagnostic information into clinically relevant risk stratification.

Liu and Dong extended this concept by incorporating inflammatory and immune biomarkers into ML-based prediction [5]. In a retrospective cohort of 340 women, they compared logistic regression, random forest, and XGBoost models using baseline clinical variables together with interleukin-33 (IL-33), C-reactive protein (CRP), and lymphocyte subset counts. XGBoost showed the best validation performance, with an area under the curve (AUC) of 0.85, accuracy of 88.1%, sensitivity of 82.4%, specificity of 88.7%, and a notably high negative predictive value (NPV) of 98.7% [5]. Importantly, however, the same model achieved a positive predictive value (PPV) of only 28.6%, reflecting the approximately 25% RPL prevalence in the source cohort and implying substantial over-referral risk if the model were applied without further calibration in routine practice; this contrast between high NPV and modest PPV illustrates how discriminatory performance metrics can mask limited clinical utility when prevalence is moderate and PPV becomes the binding constraint on decision-making [5]. Consistent with this immune-informed approach, Wu et al. developed ML-based warning models for miscarriage in 565 pregnant patients with immune abnormalities; an XGBoost model achieved the best performance (AUC 0.9209), and SHapley Additive exPlanations (SHAP) identified medication-related contributors such as the total number of medications and the use of aspirin and low-molecular-weight heparin [18]. Taken together, these studies suggest that AI may help identify clinically meaningful immune-inflammatory signatures and may improve RPL risk estimation, although further validation is still required. Key methodological characteristics of the principal AI studies discussed in this review, including their discrimination performance, validation strategy, and reported limitations, are summarized in Table 2.

These ML-derived inflammatory signatures align with mounting non-AI evidence that chronic endometritis is consistently associated with reproductive failure including RPL [25], and that recent ML-driven single-cell analyses of decidual tissue have begun to implicate neutrophil-mediated immune dysregulation, including TNF-α-polarized neutrophil phenotypes and APP–CD74 signaling, in recurrent miscarriage pathogenesis [26]. The convergence of routinely accessible inflammatory markers (CRP, IL-33, lymphocyte subsets), histopathologic findings (chronic endometritis), and ML-nominated cell-type-specific signatures supports the integration of immune-inflammatory variables into RPL prediction models.

The biological plausibility of the ML-derived inflammatory features above deserves explicit appraisal, since the clinical utility of ML signatures ultimately depends on whether they capture causal biology rather than incidental correlations. C-reactive protein (CRP) is best understood as a downstream marker of systemic low-grade inflammation rather than a direct mediator of pregnancy loss; its elevation in RPL likely reflects underlying chronic endometritis, autoimmunity, or metabolic comorbidity, and its ML utility is therefore mainly as a non-specific indicator of an inflammatory phenotype rather than as a pathogenic effector. Interleukin-33 (IL-33), by contrast, has direct experimental support for a causal role at the maternal–fetal interface: IL-33 is an alarmin released by decidualizing human endometrial stromal cells that signals through the membrane-bound ST2L receptor and is opposed by the soluble decoy receptor sST2, and Salker et al. showed that the temporal IL-33/ST2L/sST2 sequence is disordered in primary decidualizing cells from women with RPL, prolonging the implantation window and predisposing to subsequent pregnancy failure in a mouse transfer model [27]. An imbalance of the IL-33/ST2–AXL–efferocytosis axis has subsequently been shown to drive M1 polarization and dysfunctional clearance of apoptotic cells by decidual macrophages, providing a second, mechanistically distinct experimental route by which the IL-33 signal nominated by ML models is connected to pregnancy loss [28]. The ML identification of IL-33 dysregulation in RPL [5] is therefore biologically congruent with independent experimental work rather than a purely correlational signal.

Lymphocyte subset variation in RPL has a similarly well-developed mechanistic foundation that bears directly on the interpretation of ML signatures. Uterine natural killer (uNK) cells regulate decidual vasculature and extravillous trophoblast invasion, and their allorecognition of fetal trophoblast HLA-C is now considered a key determinant of placentation success rather than an artifact of immune surveillance [29]; the failure of this regulatory function, rather than the absolute number of peripheral NK cells, is what disrupts trophoblast invasion and spiral artery remodeling. CD4+CD25+FoxP3+ regulatory T cell (Treg) deficiency and a Th1/Th17-biased cytokine milieu have been independently linked to RPL through documented loss of maternal tolerance to fetal trophoblast and increased pro-inflammatory cytotoxicity at the maternal–fetal interface, and a systematic review and meta-analysis confirmed an association between altered peripheral NK cell parameters and recurrent miscarriage despite uncertainty about clinical utility [30]. ML signatures based on routine lymphocyte counts are therefore plausibly capturing this biology, although the use of peripheral blood subsets as a surrogate for decidual immune composition is a known and important limitation of clinical ML models in this area [5,29]. In our view, the IL-33 and lymphocyte-subset components of the Liu and Dong model [5] are biologically more interpretable as candidate effectors of trophoblast dysfunction and immune dysregulation, whereas the CRP component is best read as a non-specific inflammatory marker; this asymmetry should be reflected in how clinicians weigh model output.

Overall, clinical data-driven ML models currently appear to represent one of the most clinically accessible branches of AI research in RPL, particularly for clinics seeking decision-support tools based on routinely collected variables.

## 4. Imaging, Radiomics, and Embryo-Based Prediction

Imaging is central to reproductive medicine and provides biological information relevant to both miscarriage prediction and ART planning. In early pregnancy, ultrasound can capture gestational sac morphology, embryonic development, fetal cardiac activity, blood flow, and endometrial characteristics. In ART settings, embryo imaging and time-lapse systems provide information even before implantation [7,9,10,11,12,13,16,17,18,19,20]. AI methods are particularly attractive in this setting because they can extract subtle quantitative features that may be difficult to assess consistently by human observers.

### 4.1. Endometrial Imaging and Radiomics in RPL

A particularly important development is the use of radiomics to assess endometrial receptivity. Yan et al. evaluated 346 women with unexplained RPL and 369 controls and extracted radiomic features from grayscale and shear wave elastography images obtained during the window of implantation [7]. Among five ML classifiers, the combined radiomic-clinical XGBoost model showed the best performance, with an AUC of 0.871 in the training cohort and 0.844 in the testing cohort [7]. SHAP analysis further identified both imaging-derived and clinical contributors to prediction, thereby improving interpretability [7].

This study is notable because it moves beyond conventional endometrial thickness or Doppler metrics and suggests that AI-assisted radiomics may help define biologically meaningful endometrial phenotypes in women with unexplained RPL [7]. In the context of infertility treatment, such approaches may be relevant to embryo transfer planning, receptivity-oriented assessment, and individualized reproductive management.

Independent radiomic work supports this direction. Huang et al. reported an ML pipeline using radiomic features extracted from transvaginal ultrasound to optimize endometrial receptivity evaluation in women with unexplained RPL, with discrimination performance consistent with the Yan et al. cohort [31]. A subsequent extension by the Yan group moved from handcrafted radiomic features to deep-learning-enhanced ultrasound analysis combined with structured clinical variables for automated assessment of endometrial receptivity in the same RPL screening context, with the image-derived deep-learning component contributing most to fusion performance [8]. Collectively, these reports indicate that quantitative imaging of the endometrium during the implantation window can be standardized through AI in a way that is reproducible across centers and accessible from routinely acquired transvaginal ultrasound.

### 4.2. Early Pregnancy Ultrasound and Miscarriage Prediction

AI-based analysis of early pregnancy ultrasound has also shown promise for miscarriage prediction more broadly. Wang et al. developed a convolutional neural network (CNN) using 2196 ultrasound images from 1098 women with singleton pregnancies between 6 and 8 weeks of gestation [16]. Their model predicted spontaneous miscarriage with an AUC of 0.857 in retrospective testing and 0.885 in prospective validation, outperforming manual ultrasound characteristics such as crown-rump length and fetal heart rate [16]. Although this study was not specific to RPL, it demonstrates the feasibility of DL-based miscarriage prediction from early pregnancy imaging and suggests that automated ultrasound analysis may have a role in early follow-up strategies.

### 4.3. Embryo-Based Prediction in ART

A particularly relevant bridge between miscarriage prediction and ART is embryo-based prediction before or around the time of transfer. Amitai et al. used time-lapse images of preimplantation embryo development in IVF/ICSI cycles to predict first-trimester miscarriage using ML [17]. Based on a minimal subset of six morphodynamic features, their model achieved an AUC of 0.68–0.69 [17]. In a related approach, Chavez-Badiola et al. applied an AI embryo-selection assistant (ERICA) to static blastocyst images from single-embryo-transfer cycles with a positive biochemical pregnancy test and examined its ability to predict first-trimester spontaneous abortion, suggesting that morphometric embryo grading may carry prognostic information beyond implantation [19]. Moving from the embryo to the transfer cycle, Liu et al. analyzed 1664 single vitrified-warmed blastocyst transfer cycles and found that ensemble ML models, particularly a voting classifier, outperformed logistic regression in predicting early miscarriage, with maternal and paternal age, endometrial thickness, blastocyst quality, and ovarian stimulation parameters emerging as key predictors [20]. Although predictive performance across these embryo-level studies remains modest, they are conceptually important because they suggest that miscarriage risk may be encoded, at least in part, in embryo developmental dynamics and morphometry observable at or before the preimplantation stage.

Taken together, these imaging-based studies indicate that miscarriage prediction need not be confined to clinical history alone. AI may help connect embryo quality, endometrial receptivity, and early gestational development within a more unified reproductive framework.

The broader embryo-selection AI literature, while not focused on miscarriage per se, is directly relevant because optimized embryo selection in ART has the potential to reduce subsequent pregnancy loss. Foundational work demonstrated that deep convolutional neural networks can robustly classify blastocyst quality from microscopy images [32], and subsequent multi-clinic studies showed that time-lapse-trained AI models generalize across laboratories [33]. The randomized, double-blind noninferiority trial reported by Illingworth and colleagues in 2024 represents an important inflection point: deep-learning-based embryo selection was noninferior to manual morphology-based selection performed by experienced embryologists, providing the first large-scale prospective evidence for AI in this setting [24]. Scoping reviews of deep learning on time-lapse imaging confirm rapid maturation of this field [34], while pragmatic algorithms designed to fit current IVF laboratory workflows—including laboratories without time-lapse incubators—broaden applicability [35]. Pre-clinical validation of DL grading systems integrated with preimplantation genetic testing for aneuploidy (PGT-A) cycles further suggests that AI may complement, rather than replace, genetic screening [36].

## 5. Omics and Emerging Multimodal Approaches

Omics-based approaches, including genomics, transcriptomics, epigenomics, proteomics, metabolomics, immunophenotyping, and microbiome profiling, offer a biologically rich perspective on RPL pathogenesis [9]. These data are particularly attractive for AI applications because they are high-dimensional, interdependent, and not readily captured by conventional linear statistical frameworks [9]. In principle, AI-assisted omics analysis could improve mechanistic understanding, identify novel biomarkers, and support biologically informed subtyping of RPL.

However, AI studies based on omics in RPL remain relatively immature compared with clinical or imaging-based models [9]. Many are exploratory, sample sizes are limited, and external validation is uncommon [2,9]. In addition, omics data acquisition may be invasive, costly, and difficult to standardize across institutions. For these reasons, omics-based prediction has not yet reached the same level of translational readiness as routine clinical data-driven ML or ultrasound-based radiomics.

Despite this overall immaturity, several recent ML- and DL-driven omics studies have begun to nominate specific molecules and genes whose dysregulation may underlie RPL pathogenesis, providing the kind of mechanistic insight that purely clinical or imaging-based models cannot offer [37,38]. Read together, these studies suggest a small number of convergent biological themes—immune dysregulation, defective decidualization, and altered macrophage support at the maternal–fetal interface—rather than disconnected single-gene findings.

For example, using transcriptomic data, Wei et al. combined weighted gene co-expression network analysis with LASSO, support vector machine recursive feature elimination, and random forest, and identified WBP11, ACTR2, and NCSTN as decidual diagnostic biomarkers; their combined artificial neural network achieved an AUC of 1.00 in the test partition and 0.74 in an independent validation set, with RT-qPCR confirming reduced WBP11 and ACTR2 and increased NCSTN expression in RPL [37]. Luo and Zhou, applying a similar pipeline to two GEO datasets, converged on a partially distinct gene set (ZNF90, TPT1P8, FGF2, FAM166B) and demonstrated by CIBERSORT deconvolution that RPL samples exhibit higher monocyte and lower T-cell infiltration than controls [38]. Zhao et al. extended these immune findings to macrophage polarization and intercellular communication at the maternal–fetal interface in unexplained spontaneous abortion [39]. Although the hub-gene panels differ across studies, the implicated biology converges on cytoskeletal remodeling (ACTR2), Notch signaling (NCSTN, a γ-secretase component), growth factor expression (FGF2), and macrophage–decidual cell interactions.

These molecular findings are biologically consistent with the imaging-based observations described in Section 4.1: the SHAP-attributed importance of shear-wave elastography stiffness features in endometrial receptivity scoring [7] is congruent with the extracellular-matrix remodeling and decidualization defects implicated by the omics studies above. They also intersect with the immune-inflammatory variables shown to be informative in clinical models, including IL-33, CRP, and lymphocyte subset profiles [5,18], and are framed within the broader single-cell architecture of the maternal–fetal interface delineated by Vento-Tormo et al. [40]. Thus, ML applied to omics is not only generating classifiers but providing interpretable, cell-type-resolved mechanistic hypotheses—several of which involve molecules already accessible to routine clinical measurement and may therefore be tractable for translation.

However, the biological plausibility of the AI-nominated genes is heterogeneous and warrants explicit critical appraisal rather than uncritical adoption, because not every gene identified by an ML feature-selection pipeline is necessarily a causal effector of RPL. Among the Wei et al. [37] panel, ACTR2 (also known as ARP2) is a core component of the Arp2/3 complex that nucleates branched actin filaments and supports the cytoskeletal remodeling required for extravillous trophoblast (EVT) migration and invasion; failure of EVT-mediated spiral artery remodeling is an established pathogenic mechanism for early pregnancy loss and other adverse pregnancy outcomes [41]. A reduction in decidual ACTR2 in RPL, as observed by Wei et al. on transcriptomic data and confirmed at the protein level by RT-qPCR [37], is therefore biologically congruent with a recognized failure of trophoblast invasion rather than a coincidental association. NCSTN encodes nicastrin, an obligate component of the γ-secretase complex that activates Notch receptors; Notch1 is required for the development and expansion of the EVT lineage in the human placenta, and Notch family signaling has been mechanistically implicated in trophoblast endovascular invasion and arterial remodeling such that defective Notch activity has been linked to inadequate spiral artery transformation in preeclampsia and to pregnancy loss [42,43]. The ML-detected increase in decidual NCSTN expression in RPL [37] is therefore plausibly connected to a well-characterized pathway in placentation, although whether the ML signal reflects compensatory upregulation or a primary pathogenic event will require functional follow-up.

Within the Luo and Zhou [38] panel, FGF2 has the best-established mechanistic backing: basic fibroblast growth factor 2 drives decidual angiogenesis, trophoblast proliferation, and the establishment of an adequate uteroplacental vascular bed, and its dysregulation has been documented in both preeclampsia and early pregnancy loss in independent experimental and clinical reports [44]. By contrast, WBP11 (a WW-domain-binding spliceosomal component), ZNF90 (a zinc-finger transcription factor), TPT1P8 (a translationally controlled tumor protein pseudogene), and FAM166B currently lack independent experimental data on their roles in pregnancy biology. The Wei et al. [37] and Luo and Zhou [38] findings for these latter molecules should therefore be interpreted as hypothesis-generating ML signals that are statistically associated with RPL transcriptomic phenotypes in available GEO datasets, but whose causal relevance to RPL has not been independently established. Similarly, the macrophage polarization and intercellular communication networks identified by Zhao et al. [39] at the maternal–fetal interface are biologically credible in light of independent evidence on decidual macrophage function in efferocytosis and pregnancy maintenance [28], but their specific molecular hubs require confirmatory in vivo and in vitro work.

This critical reading reframes the relationship between ML output and translational claims. The appropriate epistemic posture, in our view, is a four-step appraisal cycle for any AI-nominated molecule in RPL: (i) what does the ML model actually identify, and at what discrimination and validation performance? (ii) is the candidate biologically plausible in light of independent experimental data on trophoblast invasion, decidualisation, immune tolerance, or placental vasculogenesis? (iii) is independent confirmatory evidence available (knockout/knockdown, in vivo models, prospective human studies)? and (iv) what is therefore the residual hypothesis-generating versus confirmed status of the finding? For molecules with strong independent mechanistic backing in this review—ACTR2 (cytoskeletal remodeling and trophoblast invasion [41]), NCSTN (Notch-mediated EVT development [42,43]), FGF2 (decidual angiogenesis [44]), and the IL-33/ST2 axis discussed in Section 3 [27,28]—AI offers a data-driven prioritization that converges with existing biology and may accelerate translational evaluation. For AI-nominated molecules without independent experimental support (WBP11, ZNF90, TPT1P8, FAM166B), ML provides a hypothesis-generating starting point that requires confirmatory laboratory work before clinical claims can be sustained. We have refrained from designating any AI-detected gene as a definitive “causal gene for RPL” on the basis of ML output alone; the responsible framing is that ML accelerates discovery of candidate molecules that must then be validated through conventional experimental approaches.

Nevertheless, the potential of multimodal integration remains considerable. Combining clinical history, immune-inflammatory biomarkers, ultrasound phenotypes, embryo-related metrics, and omics profiles may provide a more complete representation of reproductive failure than any single modality alone [2,9,14]. Such integrated approaches may be especially valuable in infertility care and ART, where decision-making is inherently multifactorial.

## 6. Relevance to Infertility Treatment and ART

The relevance of AI-based miscarriage and RPL prediction becomes clearer when considered within the broader context of infertility treatment and ART. Reviews of ML in assisted reproduction have highlighted applications in IVF/ICSI outcome prediction, embryo ranking, embryology laboratory workflow, and treatment personalization [10,11,12,13,14]. Dimitriadis et al. emphasized that AI may reduce subjectivity in embryo assessment and improve consistency in the embryology laboratory [11]. Salih et al. further showed that AI consistently outperformed embryologist-based assessment in studies of embryo morphology and clinical outcome prediction, while also emphasizing the need for external validation and endpoints more closely linked to ongoing pregnancy or live birth [12].

This broader ART literature is directly relevant to miscarriage prediction. If AI can better characterize embryos, endometria, and treatment response, it may help predict not only whether implantation will occur but also whether pregnancy will continue successfully. In this sense, miscarriage prediction may be viewed as part of a broader precision reproductive medicine framework rather than as an isolated application [7,9,10,11,12,13,14,17,18,19,20].

From a clinical perspective, AI may support infertility care at several levels. First, it may improve preconception counseling by helping clinicians distinguish women with relatively low residual risk from those who may benefit from expanded evaluation before treatment initiation [5,6,9]. Second, it may refine the diagnostic work-up before IVF or embryo transfer, particularly in women with previous losses or suspected multifactorial reproductive failure [6,15]. Third, imaging-based AI may help optimize endometrial assessment and embryo transfer planning, especially in women with unexplained RPL or implantation-related concerns [7]. Fourth, embryo-based prediction models may eventually contribute to risk-informed embryo prioritization, although the current evidence remains preliminary and should not yet guide routine clinical decisions [17,19,20].

AI may also inform treatment personalization beyond embryo selection, including ovarian stimulation planning. AlSaad et al. reviewed AI models for predicting ovarian stimulation outcomes in IVF and showed that the field is expanding rapidly, although most models are still based on single-center, nonpublic datasets [14]. Reviews focused specifically on ovarian stimulation similarly conclude that AI-guided gonadotropin dosing and trigger-day decisions can improve efficiency and outcomes, although large prospective trials are still needed [45]. This broader trajectory suggests that future AI systems in ART may integrate ovarian response, embryo development, endometrial receptivity, and miscarriage risk into a single decision-support framework.

A clinically important intersection of RPL care and ART is preimplantation genetic testing for aneuploidy (PGT-A), since embryo aneuploidy is a major contributor to sporadic and recurrent miscarriage. A 2025 systematic review and meta-analysis by Mumusoglu et al. reported that PGT-A in unexplained RPL was associated with a reduced clinical pregnancy loss rate (OR 0.42, 95% CI 0.27–0.67), although live-birth rate per transfer of euploid blastocysts was comparable between RPL and non-RPL patients [46]. A separate systematic review of PGT-A in recurrent reproductive failure reached similar conclusions [47]. AI-based morphologic or morphokinetic prediction of ploidy from time-lapse data is increasingly explored as a noninvasive complement to PGT-A workflows, with the potential to triage embryos for biopsy and to reduce the invasiveness and cost of genetic testing [24,34,36]. For RPL patients, the combined use of optimized embryo selection, PGT-A integration, and AI-supported endometrial receptivity assessment may therefore represent a synergistic translational strategy.

The broader contemporary appraisal of AI in ART is appropriately cautious. Early surveys at major reproductive-medicine conferences documented rapid expansion of AI applications across the IVF workflow but also highlighted gaps in standardization and reproducibility [48]. More recent commentary explicitly distinguishes the “dream” of AI-driven precision reproductive medicine from the current reality of largely retrospective, single-center proofs of concept [49], and questions whether ART is yet ready for prime-time AI deployment outside research settings [50]. These appraisals, taken together, support a pragmatic stance in which AI is incorporated into ART care selectively, in well-defined tasks with adequate validation, rather than across the workflow indiscriminately.

### Mapping AI Applications onto the ART Clinical Pathway

A clinically oriented way to assess the relevance of AI to RPL is to map AI applications onto the ART pathway: preconception work-up, ovarian stimulation, embryo selection, endometrial receptivity assessment, embryo transfer, early-pregnancy monitoring, and live birth. At the work-up stage, ML applied to expanded diagnostic panels has the potential to refine risk stratification beyond the limited features endorsed by current guidelines, as demonstrated by the gap between the 81.86% balanced accuracy of the full Bruno feature set and the 58.52% achieved with ESHRE features alone [6]; for couples with previous loss this could rationalize which investigations to prioritize. At the ovarian-stimulation stage, ML models for gonadotropin dosing, follicular monitoring, and trigger-day selection [14,45,50] may indirectly reduce miscarriage risk by improving oocyte cohort quality and avoiding suboptimal cycle outcomes, though direct evidence linking stimulation-AI to live birth in RPL populations is currently lacking.

At embryo selection, AI is the most mature application: the 2024 noninferiority randomized controlled trial of Illingworth et al. demonstrated that DL-based selection is noninferior to manual morphology by experienced embryologists [24], with foundational [32,33] and pragmatic [34,35] work supporting generalizability. However, the trial endpoints were predominantly clinical pregnancy and ongoing pregnancy, not miscarriage after detection of fetal cardiac activity (FHB+) or live birth specifically; data on whether AI selection differentially reduces post-FHB miscarriage in RPL populations are still required. Embryo aneuploidy, the single most important embryonic contributor to sporadic and recurrent miscarriage, sits at the intersection of AI and PGT-A: meta-analyses suggest that PGT-A in unexplained RPL reduces clinical pregnancy loss while leaving per-euploid-blastocyst live-birth rates comparable across populations [46,47], and AI-based noninvasive ploidy prediction is being explored as a complement to invasive biopsy [24,34,36]. At the endometrial-receptivity stage, radiomics and DL-based ultrasound analysis [7,8,31] target a determinant that is potentially modifiable in the same cycle (window of implantation timing, hysteroscopic evaluation for chronic endometritis [25], correction of subclinical inflammation), and is therefore a particularly actionable insertion point for AI in RPL care.

After transfer, AI-based analysis of early pregnancy ultrasound [16] and ML for post-FET early-miscarriage prediction [20] address the early-pregnancy-monitoring stage, where the relevant endpoints become miscarriage after FHB+, ongoing pregnancy beyond the first trimester, and live birth. Throughout this pathway, paternal and male-factor variables (sperm parameters, paternal age, advanced paternal-age effects on aneuploidy) and sperm-selection AI are increasingly recognized as missing dimensions of most current RPL models and deserve incorporation in future work [12,48]. Considered as a sequence, the ART pathway clarifies why isolated improvements in a single AI tool (for example, embryo grading) cannot by themselves resolve RPL care, and why integration across stages, with endpoints anchored on live birth rather than implantation or biochemical pregnancy, is the more meaningful target for clinical AI in this population.

Importantly, however, the current evidence does not justify strong clinical claims. Most available models have not been prospectively tested in real-world IVF settings for their ability to improve live birth outcomes, and many remain proof-of-concept tools rather than validated clinical systems [2,9,12,13,14].

## 7. Current Limitations and Barriers to Clinical Translation

Despite encouraging progress, several barriers continue to limit the routine adoption of AI in miscarriage and RPL care. Moustakli et al. highlighted major challenges including small and heterogeneous datasets, inconsistent diagnostic definitions, limited external validation, lack of prospective trials, and inadequate standardization of data collection across studies [9]. These limitations are echoed throughout the broader ART literature [10,11,12,13,14].

A recent systematic review by Imani et al. analyzed 26 studies on ML for recurrent miscarriage prediction and concluded that, although many models reported promising discrimination, the field remains constrained by a substantial reproducibility and validation gap [2]. Most studies relied on retrospective single-center datasets, external validation was rare, calibration reporting was limited, and handling of missing data was often inadequate [2]. These methodological weaknesses remain a major obstacle to clinical implementation.

Several methodological issues deserve particular attention in RPL-AI work. First, simulation studies have shown that common class-imbalance corrections such as random over- and under-sampling and SMOTE can substantially worsen model calibration, producing systematic over-estimation of risk that is not always recoverable by post hoc recalibration [51]; this finding has recently been extended to a broader range of machine-learning algorithms [52]. The implication for RPL, where individualized absolute risk estimation is the clinical goal, is that uncorrected models with appropriate evaluation metrics may be preferable to aggressively rebalanced ones. Second, broader systematic reviews of ML in pregnancy care have shown that ML methods do not consistently outperform well-specified logistic regression once analytic and reporting quality are accounted for, underscoring that algorithmic flexibility alone does not guarantee clinical utility [53]. Third, adherence to TRIPOD and PROBAST reporting standards in obstetric ML studies remains poor [54], a gap that helps explain why otherwise promising internal performance often does not generalize. RPL-focused AI research should therefore prioritize calibration reporting, decision-curve analysis, prospective and external validation, and transparent handling of missing data.

A related point is that discriminatory performance (AUC, accuracy) is conceptually distinct from clinical utility (calibration, decision-curve net benefit, predictive values at the relevant prevalence, generalizability under distribution shift, and demonstrated impact on patient outcomes), and these dimensions should be appraised separately. The Liu and Dong study illustrates this distinction concretely: AUC 0.85, accuracy 88.1%, sensitivity 82.4%, specificity 88.7%, and NPV 98.7% coexist with a PPV of only 28.6% [5]. In a population with approximately 25% RPL prevalence the latter figure is the operationally binding constraint, because most positive predictions would be false alarms; this would translate into substantial over-referral, over-investigation, and patient anxiety if such a model were deployed without explicit threshold tuning and decision-analytic justification. Reported discrimination should therefore not be read as clinical readiness, and routine reporting of calibration plots, decision-curve analyses, and PPV/NPV at clinically realistic prevalences should be an editorial expectation in this literature [51,52,53,54].

The field is also evolving toward AI-specific reporting and evaluation standards. The TRIPOD+AI statement extends transparent reporting of prediction models to machine-learning methodology, with explicit items on dataset description, preprocessing, model architecture, hyperparameter tuning, internal-external validation, fairness, and uncertainty quantification [22]. DECIDE-AI provides complementary guidance for the small-scale, live, clinical evaluation phase that precedes large-scale trials of AI systems, with attention to human factors, error analysis, and safe deployment [23]. Most currently published RPL-AI studies were conducted before these guidelines were widely adopted, and applying them retrospectively to existing models highlights why few are implementation-ready: prospective external validation, calibration after deployment, and patient-relevant outcome endpoints (live birth, miscarriage after FHB+) are largely absent. Adoption of TRIPOD+AI for development reports and DECIDE-AI for early clinical evaluation should therefore be regarded as a precondition rather than a refinement for clinical translation in this area.

Interpretability is another critical issue. In reproductive medicine, predictive outputs may influence counseling, monitoring intensity, diagnostic testing, and treatment strategy. Black-box models may therefore be difficult to integrate into practice unless they provide clinically meaningful rationale [9,13]. Lee et al. argued that more transparent “glass-box” approaches may ultimately be preferable in embryo selection and, by extension, in related applications such as RPL prediction [13]. This view aligns with earlier arguments that interpretable, rather than black-box, models should be used for embryo selection given the ethical and clinical stakes involved [55].

Ethical and regulatory challenges must also be addressed. AI systems may inherit demographic, institutional, or socioeconomic biases from their training data, potentially exacerbating inequities in access to reproductive care [9,13,14]. Transparency, reproducibility, privacy protection, fairness, and clinician oversight should therefore be regarded as essential components of future model development and implementation.

## 8. Future Directions

Future research should move toward multimodal AI systems that integrate clinical history, laboratory variables, imaging data, embryo-derived metrics, and, where appropriate, omics profiles. Such models may better reflect the complexity of reproductive failure than any single-modality approach [2,9,14]. In infertility treatment and ART, this could support individualized preconception counseling, expanded diagnostic triage, embryo transfer timing, endometrial assessment, and personalized early pregnancy surveillance. To overcome the limited dataset sizes characteristic of single-center RPL cohorts, federated learning—in which multi-institutional model training is performed without centralized sharing of identifiable patient data—is a particularly attractive route forward and is increasingly considered a foundational paradigm for digital health [56].

Prospective multicenter validation should be regarded as a priority. Future studies should increasingly assess clinically meaningful endpoints such as ongoing pregnancy and live birth rather than surrogate outcomes alone [2,12,13]. Explainable AI methods, including SHAP and related approaches, should also be incorporated whenever possible to improve clinician trust and patient-facing usability [7,13].

Ultimately, the greatest contribution of AI may not be a single superior prediction score but the development of integrated reproductive decision-support systems capable of linking implantation, miscarriage risk, and treatment response within one coherent clinical framework.

## 9. Conclusions

AI is emerging as a potentially useful tool for the prediction and stratification of miscarriage and recurrent pregnancy loss. Current studies suggest that ML may outperform limited conventional feature sets when broader clinical, immune-inflammatory, and imaging-derived data are incorporated [5,6,7]. Additional evidence from IVF and ART research indicates that AI is also being applied to embryo selection, embryology workflow, and ovarian stimulation prediction, thereby providing a translational context for miscarriage-risk modeling [10,11,12,13,14,17,18,19,20].

However, clinical implementation remains premature. Most currently available models are retrospective, internally validated only, limited by poor calibration reporting and inadequate explainability, and have rarely been evaluated against live-birth or post-FHB miscarriage endpoints rather than discrimination metrics alone [2,9,51,52,53,54]. Nevertheless, the field is evolving rapidly, and the adoption of TRIPOD+AI [22] and DECIDE-AI [23] reporting and evaluation standards, together with mapping of AI applications onto the ART clinical pathway, can be expected to accelerate the transition from proof-of-concept to clinically useful tools. With more rigorous validation, multimodal integration, interpretable design, and patient-relevant endpoints, AI may become an important component of precision reproductive medicine, potentially supporting not only miscarriage prediction but also more individualized infertility care and ART decision-making.

## Figures and Tables

**Table 1:** Operational definitions of recurrent pregnancy loss across major guidelines and selected primary AI studies cited in this review.

Source	Operational Definition	Notes/Exclusions
ASRM 2026 [3]	≥2 spontaneous pregnancy losses before 22 weeks of gestation; hCG-confirmed biochemical losses counted	Confirmed molar and ectopic pregnancies excluded
ESHRE 2022 [4]	≥2 spontaneous pregnancy losses	Earlier evaluation and intervention encouraged
Bruno et al. [6]	RPL Unit referrals stratified by number of miscarriages into 4 risk classes	Italian academic RPL Unit cohort
Liu and Dong [5]	≥2 consecutive spontaneous miscarriages before 20 weeks of gestation	Routine immune/inflammatory panel as predictors
Yan et al. [7,8]	Unexplained RPL after standard work-up	Endometrial radiomic/DL ultrasound
Imani et al. [2]	Variable across 26 included studies; heterogeneity noted	Systematic review of ML for RPL

**Table 2:** Summary of selected primary AI studies in miscarriage and recurrent pregnancy loss prediction, with reported discrimination metrics, validation strategy, and principal limitations. AUC, area under the receiver-operating-characteristic curve; bal. acc., balanced accuracy; CNN, convolutional neural network; DL, deep learning; ML, machine learning; n.r., not reported; PPV, positive predictive value; RCT, randomized controlled trial; SVM, support vector machine; XGB, XGBoost.

Study	Setting/Population	*n*	Algorithm	Discrimination	Validation	Key Reported Limitation
Bruno et al. [6]	RPL clinical work-up (Italy)	734	SVM (4-class)	81.86% bal. acc. (43 features)	Internal only (cross-validation)	Single-center; missing data; no calibration; no external validation
Liu and Dong [5]	RPL + immune/inflammatory panel	340	XGB vs. LR, RF	AUC 0.85; PPV 28.6%; NPV 98.7%	Internal (70/30 split)	Low PPV implies over-referral risk; single-center; retrospective
Yan et al. [7]	Unexplained RPL endometrial radiomics	715	XGB (radiomic + clinical)	AUC 0.871 train/0.844 test	Internal split	Single-center; calibration not detailed
Wang et al. [16]	Early-pregnancy gestational-sac US (general)	1098	CNN	AUC 0.857 retro/0.885 prosp.	Prospective cohort	Imaging only; not RPL-specific
Amitai et al. [17]	IVF/ICSI time-lapse embryo data	n.r.	ML on 6 features	AUC 0.68–0.69	Internal only	Modest performance; not RPL-restricted
Wu et al. [18]	Pregnancies with immune abnormalities	565	XGB + SHAP	AUC 0.9209	Internal only	Single-center; calibration n.r.
Illingworth et al. [24]	IVF embryo selection RCT	1066	DL vs. embryologist	Noninferior (clinical pregnancy)	Multicenter prospective RCT	Endpoint not live birth; not RPL-specific

## Data Availability

No new data were created or analyzed in this study.

## Abbreviations

AI, artificial intelligence; ART, assisted reproductive technology; AUC, area under the curve; ASRM, American Society for Reproductive Medicine; CNN, convolutional neural network; CRP, C-reactive protein; DL, deep learning; ESHRE, European Society of Human Reproduction and Embryology; IL-33, interleukin-33; IVF, in vitro fertilization; ML, machine learning; RPL, recurrent pregnancy loss; SHAP, SHapley Additive exPlanations; SVM, support vector machine.
